# Attractor-Based Obstructions to Growth in Homogeneous Cyclic Boolean Automata

**DOI:** 10.4172/jcsb.1000209

**Published:** 2015-11-04

**Authors:** Bilal Khan, Yuri Cantor, Kirk Dombrowski

**Affiliations:** 1 Department of Sociology, University of Nebraska-Lincoln, Lincoln, Nebraska, USA; 2 Department of Computer Science, The Graduate Center, City University of New York, USA

**Keywords:** Attractor-based obstructions, Cyclic boolean automata, Synchronous boolean, Robustness

## Abstract

We consider a synchronous Boolean organism consisting of N cells arranged in a circle, where each cell initially takes on an independently chosen Boolean value. During the lifetime of the organism, each cell updates its own value by responding to the presence (or absence) of diversity amongst its two neighbours’ values. We show that if all cells eventually take a value of 0 (irrespective of their initial values) then the organism necessarily has a cell count that is a power of 2. In addition, the converse is also proved: if the number of cells in the organism is a proper power of 2, then no matter what the initial values of the cells are, eventually all cells take on a value of 0 and then cease to change further. We argue that such an absence of structure in the dynamical properties of the organism implies a lack of adaptiveness, and so is evolutionarily disadvantageous. It follows that as the organism doubles in size (say from m to 2m) it will necessarily encounter an intermediate size that is a proper power of 2, and suffers from low adaptiveness. Finally we show, through computational experiments, that one way an organism can grow to more than twice its size and still avoid passing through intermediate sizes that lack structural dynamics, is for the organism to depart from assumptions of homogeneity at the cellular level.

## Introduction

The subject of cellular automata has received much attention since John Von Neumann’s seminal work [1] on the dynamics of a grid of cells which evolve in discrete time steps according to rules based on their neighbor’s values (e.g., see the surveys in ref. [2,3]). Conway’s Game of Life [4], perhaps the most famous example of cellular automata, consists of an infinite two-dimensional orthogonal grid of Boolean cells whose values are synchronously updated 1. Cellular automata are frequently studied by considering their collective dynamics. Wolfram [5], for example, examined the complexity of finding “Garden of Eden States” (i.e., states that are unreachable from any other state), as well as determining whether a network can reach a state in which all cells have value 1 (i.e., a question that is now known as the “All-Ones Problem”). Both these problems generally become computationally infeasible for all but the smallest one-dimensional networks [6].

A random graph model for automata was introduced by Stuart Kauffman in the course of his research on gene regulatory networks. These so-called “NK networks” [7] consist of *N* cells, each of which is connected to a randomly chosen subset of K cells. Kauffman and others considered self-organization and the spontaneous emergence of order [8] in NK and related networks. Consensus is a particular form of emergent order that has received particular attention, especially in the context of social systems. Miller considered consensus in the standing ovation problem as a means to examine behavior in social networks using computational models [9]. Arenas surveys network structures which lead to emergent features and reports on the implications of consensus emergence in a variety of settings [10]. In his work, the community structures of networks (i.e., clusters of densely interconnected cells, between which connections are sparse) play a crucial role. Ball describes how many natural systems rely on characteristics akin to community structure in order to reach a level of consensus robustly in the presence of noise [11]. Consensus problems are closely related to our research since both seeks to understand dynamical systems which move towards uniformity irrespective of initial conditions [12], and to identify the social network properties that lead to stasis and uniformity [13].

The organisms we consider in this paper are discrete Boolean cellular automata of the NK type, though we restrict ourselves to K=2 [14] and require that the cells be connected deterministically to form a circle. Such cyclic networks have received considerable attention themselves [6]. Like most prior research, we too (at least initially) consider only cellular automata that are homogenous at the cellular level, that is, cyclic networks in which all cells operate according to an identical update rule. For simplicity, we only consider networks in which cells synchronously update their values–recent progress in sequential dynamical systems [15-17] has shown that the behavior of more general asynchronous systems with small temporal variations can be examined by “equivalent” synchronous systems [18].

Where the All Ones Problem asks if the state in which all cells have value 1 is reachable from any other state, here we seek to determine if there is a state that is reachable from every other state. The networks we will consider are so simple as to lack community substructures, and yet always reach consensus regardless of noise. This is possible because (as we shall prove) their dynamics exhibit a single unique attractor. This work extends earlier results on thermal robustness and attractor density in synchronously updated cyclic Boolean networks [19].

## Background

In this section we introduce basic terminology concerning Boolean automata through examples, and provide some motivating context. The terminology is rendered formally later, in Section 5.

**Figure 1:**
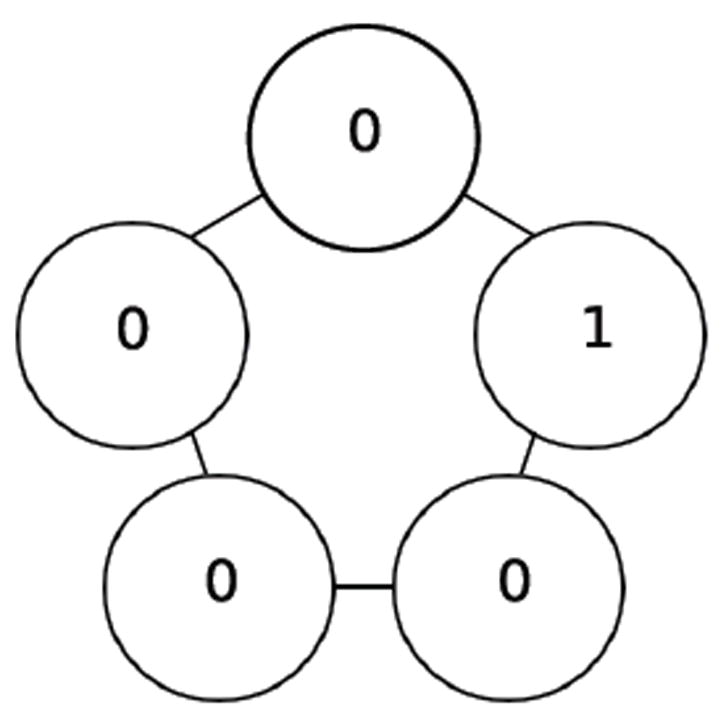
Initial state.

In this paper we investigate cyclic (i.e., circular) organisms where each cell has an instantaneous value of either 0 or 1. The organism is homogenous in that it evolves over time by having each cell repeatedly apply the same update rule. Although there are many choices of update rules, the one we consider here is XOR, which takes into consideration just the two immediate neighbors of the cell [14]. The XOR rule specifies that if the values of a cell’s two neighbors differ, then the cell takes a value of 1 at the next time step; conversely, if the values of a cell’s two neighbors agree, then the cell takes a value of 0 at the next time step). So, for example, if a cell’s two neighbors have values 0 and 1, then at the next time step, the cell will take on a value of 1—an example of such a cell appears at the top of the 5-cycle in Figure 1. On the other hand, if a cell’s left and right neighbors both have value 0, then at the next time step, the cell will take on a value of 0—an example of such a cell appears at the bottom-left of the 5-cycle in Figure 1.

**Figure 2:**
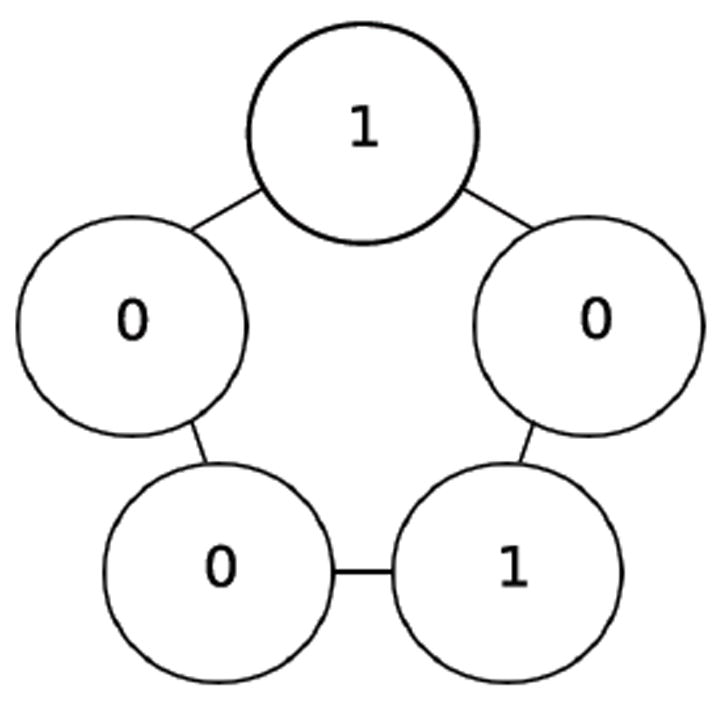
Successor state.

**Figure 3:**
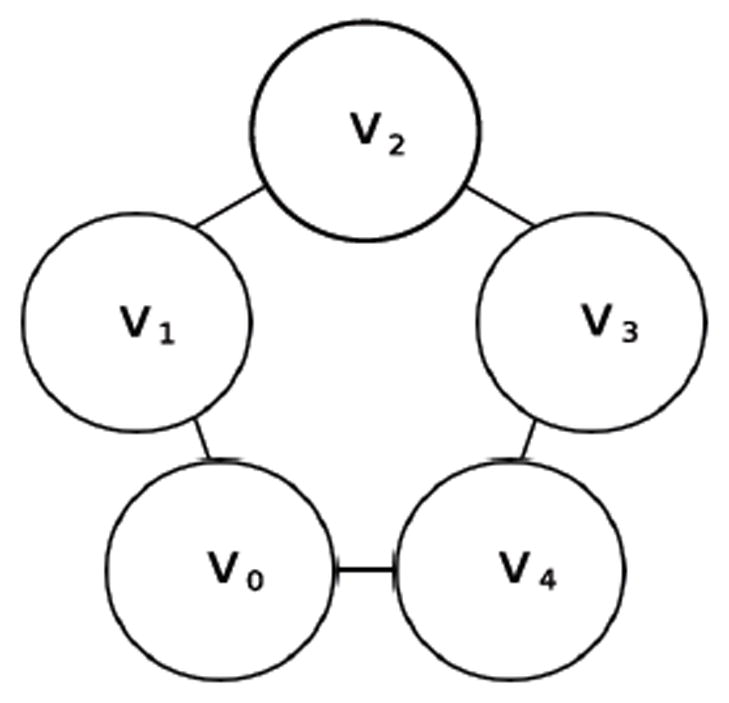
Size 5 network.

**Figure 4:**
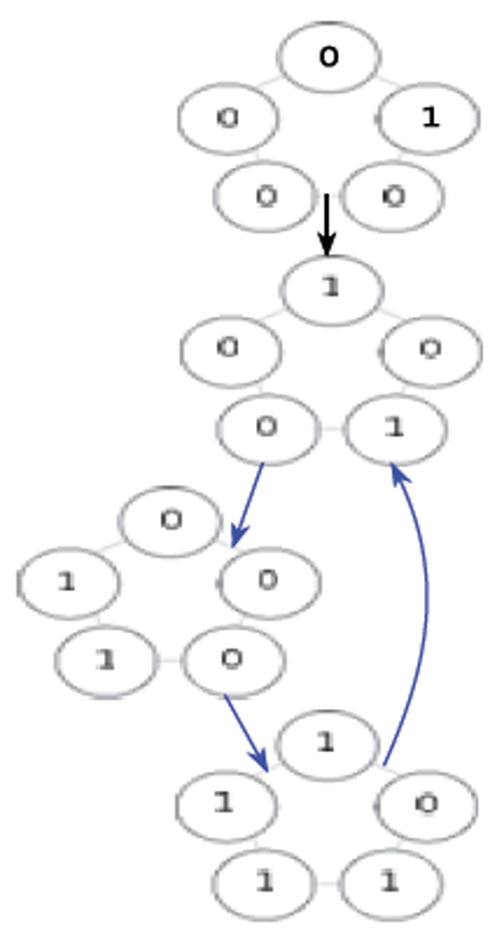
Successor states from Figure 1.

**Figure 5:**
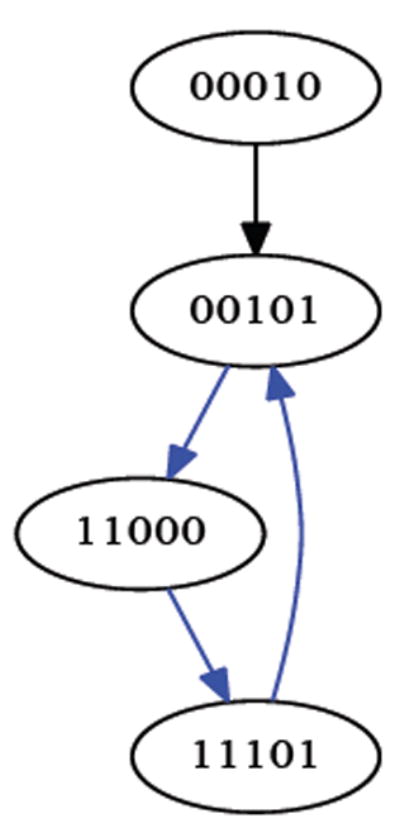
Attractor with network states.

If one synchronously (i.e., simultaneously) applies the XOR update rule at each of the cells in Figure 1 then one arrives at the configuration in Figure 2. Each of these configurations is referred to as a state. If we fix index of the organism’s cells, for example as shown in Figure 3, then the state depicted in Figure 1 can be named 00010, while the state depicted in Figure 2 can be named 00101. We say that the successor of 00010 is 00101. Following in this manner, one finds that the successor of 00101, is 11000, and the successor of 11000 is 11101 and its successor in turn is 00101. This sequence of state transitions is shown in Figures 4 and 5. Using software developed previously [19], we experimentally simulate the size 5 homogeneous XOR organism and output the dynamics as a file. From this output, we rendered Figure 5 using Graphviz [20]. If we start from the state 00101 and move forward 3 time steps, we return back at the state 00101 (since 00101→11000→11101→00101), such a structure is called an attractor. Because it takes 3 time steps to go around this example attractor once, it is said to be an attractor of length 3.

Because the organism is of finite size, and each of its cells can only take on one of two values (either 0 or 1), there are only finitely many states the organism can be in. An organism of size 5 can be in one of 2 × 2 × 2 × 2 × 2=32 different states; an organism with *N* cells can be in one of 2*^N^* different states. Taken as a collection of states, this is referred to as the state space of the organism.

**Figure 6:**
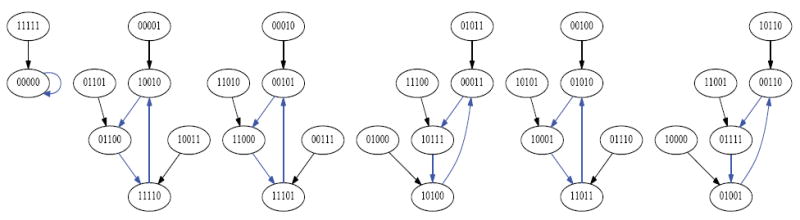
Dynamics graph of the cyclic organism of size 5, in binary.

**Figure 7:**
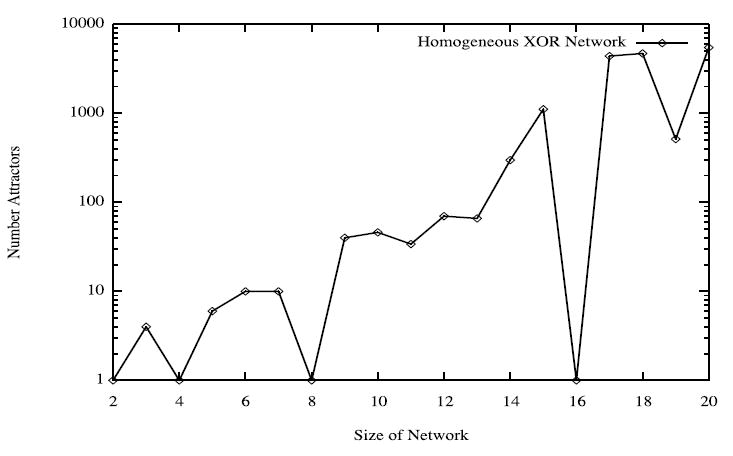
Number of Attractors vs Network Size.

Suppose we draw an arrow from one state *X* to another state Y, whenever simultaneously applying XOR at each of the cells in the organism when it is in state *X* would lead to the organism being in state Y. Then each state would have exactly one arrow emerging from it, since the application of XOR is completely deterministic. The resulting directed network would have 2*^N^* states as its vertices, and 2*^N^* arrows as its edges. Such a rendering is called the dynamics graph of the organism. Figure 6, which we create in the same manner as before by simulating the network to produce an output file of the dynamics and using Graphviz to render, shows the complete dynamics graph of the cyclic organism of size 5. We generate Figure 7 again using the same technique only this time with the binary output converted to decimal to show the same graph but vertices (states) labeled by the decimal equivalent of the binary name. From either of these diagrams, one can verify that there are 6 attractors in all. Five of these attractors have length 3, while one of them has length 1.

**Figure 8:**
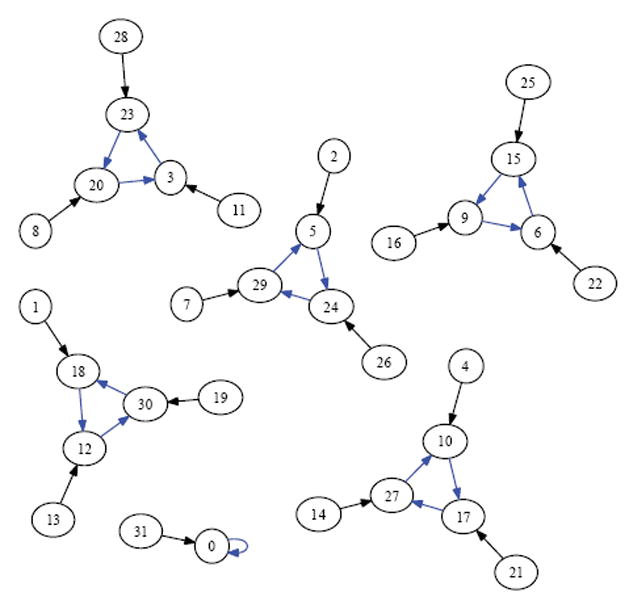
Dynamics graph of organism of size 5, in decimal.

**Figure 9:**
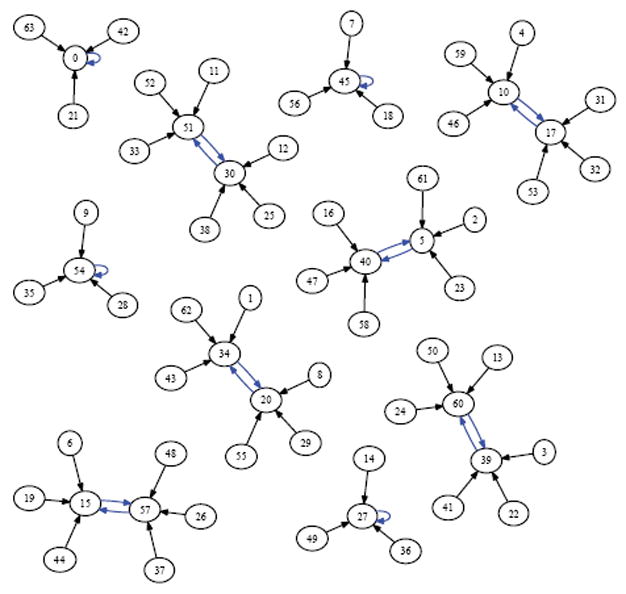
Dynamics graph of the organism of size 6, in decimal.

While the organism of size 5 has 6 attractors, it turns out the organism of size 6 has 10 attractors (4 of length 1, and 6 of length 2). This is seen in the dynamics graph of the organism of size 6, which is given in Figure 8. As before, we generate Figure 8 using Graphviz and the simulated dynamics output using our software. Indeed, as the organism grows from size 5, 6, 7, so on and onward, the number of attractors rises and falls abruptly. This is quantified in the plot in Figure 9 below, where the x-axis is the organism’s size (number of cells), and the *y*-axis of this plot is the (base 2) logarithm of the number of attractors. Figure 9 is generated using collated experimental results from simulations of homogeneous XOR networks of sizes 2 through 20 where the output attractor counts are graphed using gnuplot [21]. We see from the plot that while the general trend is for the number of attractors to grow exponentially as the organism gets larger, every so often the organism reaches a size for which the number of attractors plummets to 1—these are precisely those points where the curve passes through a point whose *y*-coordinate is *log*_2_ (1)=0. One can see again from the plot in Figure 9 that this occurs precisely when the organism reaches size 2, 4, 8, and 16.

One might conjecture from the experimental data in the above graph that whenever the cyclic organism reaches a size that is a proper power of 2 (e.g., 2, 4, 8, 16, 32, 64, …) then the number of attractors in its dynamics graph is exactly 1. The majority of this paper is devoted to proving both this statement, as well as its converse: if an organism has just one attractor in its dynamics graph, then the organism necessarily has a power of 2 many cells.

### Interpreting the number of attractors

Biological organisms are subjected to Darwinian preferential selection based on the evolutionary advantages of their properties, within the context of the ecosystem. Dynamics of Boolean networks are simpler than biological networks while possessing some essential similar properties. To the extent that the mathematical model we have presented here is an idealized rendering of a biological organism, of what significance is the number of attractors? Let us examine, at the informal level of metaphor, what the implications of having a very “large” or very “small” number of attractors have on an organism within a real-world ecosystem. We will assume that this ecosystem provides the organism with two types of signals: environmental stimuli and thermal noise.

Environmental stimuli are high amplitude signals from the ecosystem to which the organism needs to be adaptive and react. Thermal noise, on the other hand, consists of low amplitude signals against which the organism needs to be robust and not react. An organism typically is spinning inside some attractor A. When it receives a signal from the outside, one or more of its cell’s values may be perturbed (the 0’s may become 1’s and vice versa). This effectively throws the organism out of its present state within *A*, over to some other vertex *v* in it’s the dynamics graph. From v, the organism follows the arrows until it lands once again in an attractor *A*′. The attractor *A*′ may or may not be the same as the attractor *A*.

For example, if a size 5 organism (see Figure 7) is in the attractor *A*=15→9→6→15 and receives a stimulus, the organism might get thrown to state *v*=28, which case it would land in the attractor *A*′=23→20→3→23. In this case *A* /= *A*′. If however, the stimulus resulted in the organism being thrown into state *v*=22, then it would land in the attractor *A*′ =15→9→6→15. In this case *A*=*A*′. From combinatorial arguments alone then:

- In an organism which has a very large number of attractors, *A*′ will almost never be the same as *A*. This makes the organism very adaptive (because almost any stimulus will cause it to leave its present attractor), but not very robust (for the same reason). Even the slightest amount of thermal noise will cause the organism to jump into a different attractor. The organism has low robustness.
- In an organism which has a very small number of attractors, *A*′ will almost always be the same as *A*. This makes the organism very robust (because almost no stimulus will cause it to leave its present attractor), but not very adaptive (for the same reason). Even the greatest amount of environmental stimulus will not cause the organism to jump into a different attractor. The organism has low adaptiveness.

An alternate interpretation of attractor counts comes to us from the world of neural networks. If the organism is viewed as a set of interconnected neurons, then the state space of that organism can be interpreted as a mental space. Following this analogy, the dynamics of the state space are mental processes and those individual processes each lead to an attractor. The formalism of attractors has been used to describe memories in the brain [22,23]. The neural network cycles infinitely through the states of an attractor, until perturbed through stimulus by some outside force, introducing a change in dynamics. A mental process that has this property of persisting over time until disturbed by an outside force is a memory or a thought. The number of distinct memories or thoughts a network can represent reflects the quantity of information that the network can process or store. Attractors and their potential for information storage has received much attention especially in the areas of neural networks and neural science. The number and length of attractors have been studied extensively [8,24-28]. In ref. [29], simple dynamic systems are studied for their large number of attractors and the ease that they can be manipulated to perform neural processes.

It is clear then that to attain a reasonable trade-off between being robust and being adaptive, the organism requires an “intermediate” number of attractors. Having too many attractors implies a loss of robustness, and having too few means a loss of adaptiveness. What is too many and too few? We know that for an organism of size N, the number of states in the state space is 2*^N^*, so this is an upper bound on the number of attractors. Since every state leads to another state and the total number of states is finite, there has to be at least one attractor in the dynamics graph; thus 1 is a lower bound on the number of attractors.

Given the above observations, the main result of this paper, that whenever the cyclic organism reaches a size that is a proper power of 2 (e.g., 2, 4, 8, 16, 32, 64, …) the number of attractors in its dynamics graph is exactly 1, implies that every time an organism doubles in size, it necessarily passes through a size at which it has very low adaptiveness. In the next section of this paper, we show through computational experiments, that one way to remedy this low adaptiveness is for the organism to depart from homogeneity at the cellular level. These observations may reflect underlying evolutionary forces driving morphogensis and cellular differentiation.

## Avoiding low adaptiveness by departing from homogeneity

We explore whether low adaptivity can be avoided by introducing cellular differentiation. In the homogeneous network every cell applies the same update rule. We define a minimally heterogeneous organism to have cellular differentiation at a single cell meaning that only at this one cell is a different update rule applied. Experimentally, we simulate the state space dynamics of every possible minimally heterogeneous organism at sizes 2, 4, 8, and 16. For larger sizes 32, 64, 128, and 256 where complete simulation of the state space dynamics is computationally intractable, we sample the state space. To sample the state space, we randomly select 1000 initial states and successively compute their successor states until we find an attractor and record each attractor discovered. The number of attractors discovered by sampling provides a lower bound for the total number of attractors.

A cell’s update rule takes as input the Boolean values of its neighbours. We refer to the neighbor to left *v_i_* × 1 and the neighbor to the right as *v_i_*+1 where i refers to the index of cell applying the rule. For example, the state in Figure 1 can be indexed according to Figure 3. If we let *i*=2, then *v*_*i*-1_=*v*_1_ and *v_i_*_+1_=*v*_3_. Then we can determine that the value of the neighboring cells is 0 at *v_i-1_* and 1 at *v*_*i*+1_. Since there are 2 neighbors and each can have one of two possible values 0 or 1, then there are 2*2 or 4 possible input combinations of neighbor values. A cell’s value must be either 0 or 1 as a result of each of the possible the inputs from its neighbors. Since there are 2 choices for each of the 4 possible input combinations, there are 24 or 16 possible rules. In Table 1 we define these rules using Boolean logic. The rules are ordered in ascending order according to the Boolean value of the input values for which the rule sets the cell’s value to have the value 1.

In Table 2, we see the results of the simulation. At increasing sizes which are proper powers of 2, minimally heterogeneous organisms where the single cell applies one of following rules {1, 3, 5, 7, 8, 10, 12 and 14} have an increase in number of attractors. Note, in Table 2 each column corresponds to one of these minimally heterogeneous organisms. For networks of sizes over 24, exhaustively enumerating the entire dynamics graph is computationally intractable [30-33]. The break in Table 2 between N=16 and N=32 corresponds to a shift in computational strategy, from finding the exact number of attractors by fully exploring the dynamics graph, to finding a lower bound of number of attractors by sampling the dynamics graph starting at 1000 random initial start states. The minimally heterogeneous organisms where the single cell applies one of the following rules {0, 2, 4, 9, 11, 13 and 15} are not shown in Table 2. For these minimally heterogeneous organisms cellular differentiation did not circumvent the collapse to a single attractor.

**Figure 10:**
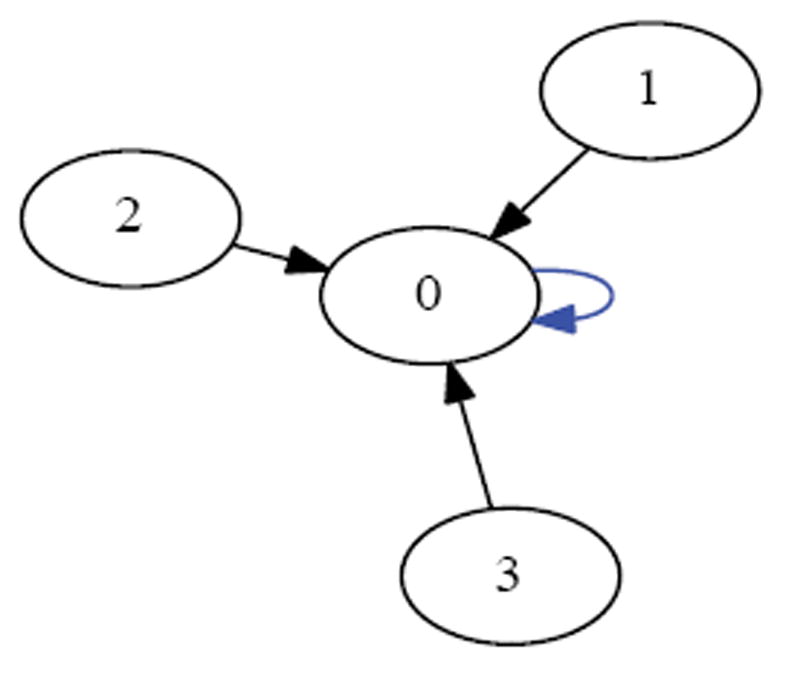
XOR organism size 2.

**Figure 11:**
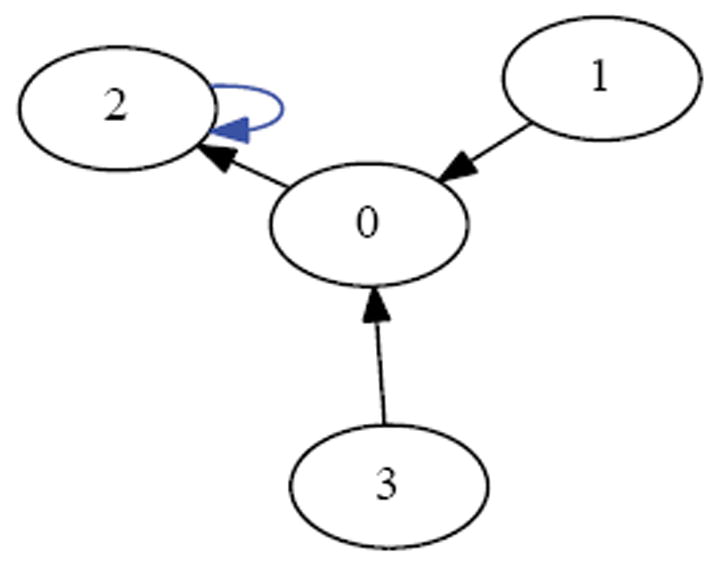
Minimally heterogeneous size 2 XOR+Rule 1.

**Figure 12:**
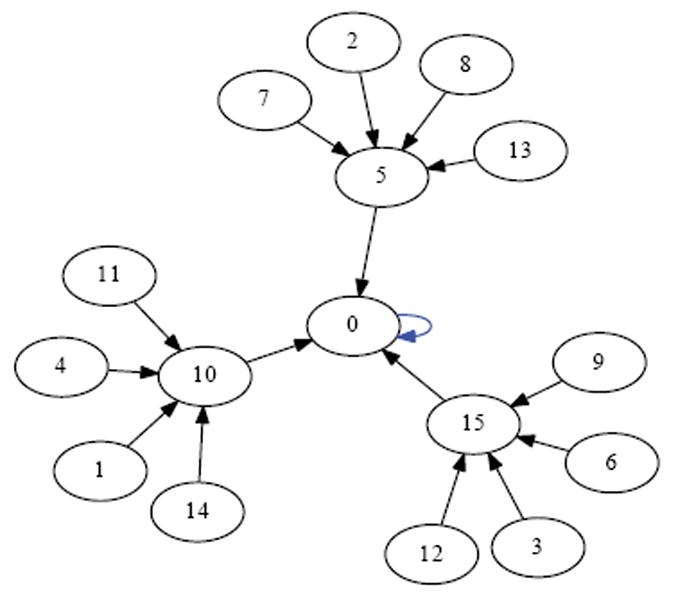
XOR organism size 4.

**Figure 13:**
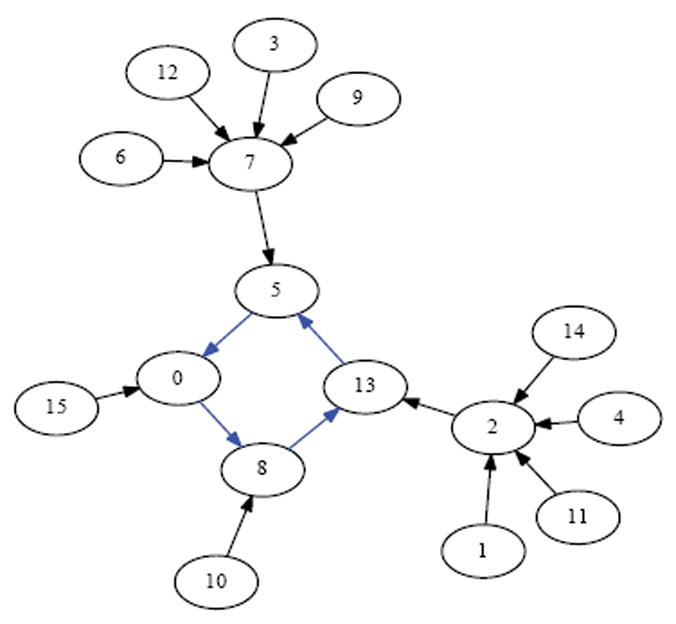
Minimally heterogeneous size 4 XOR+Rule 1.

**Figure 14:**
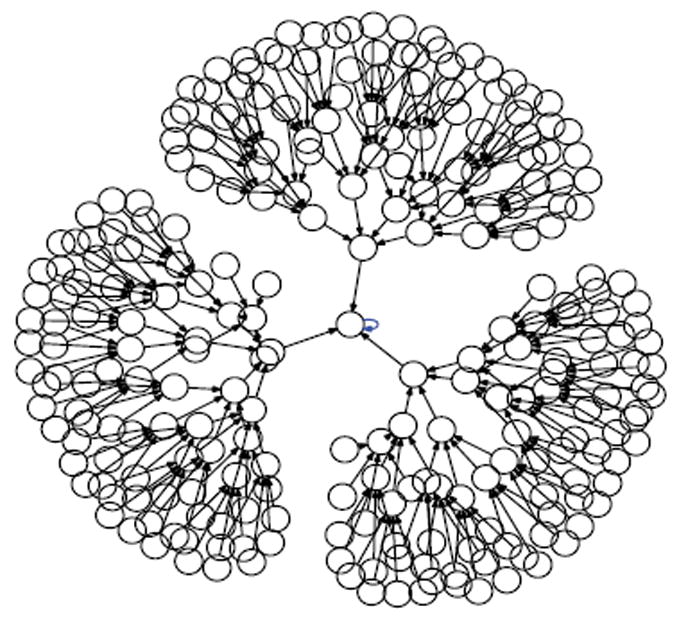
XOR organism size 8.

**Figure 15:**
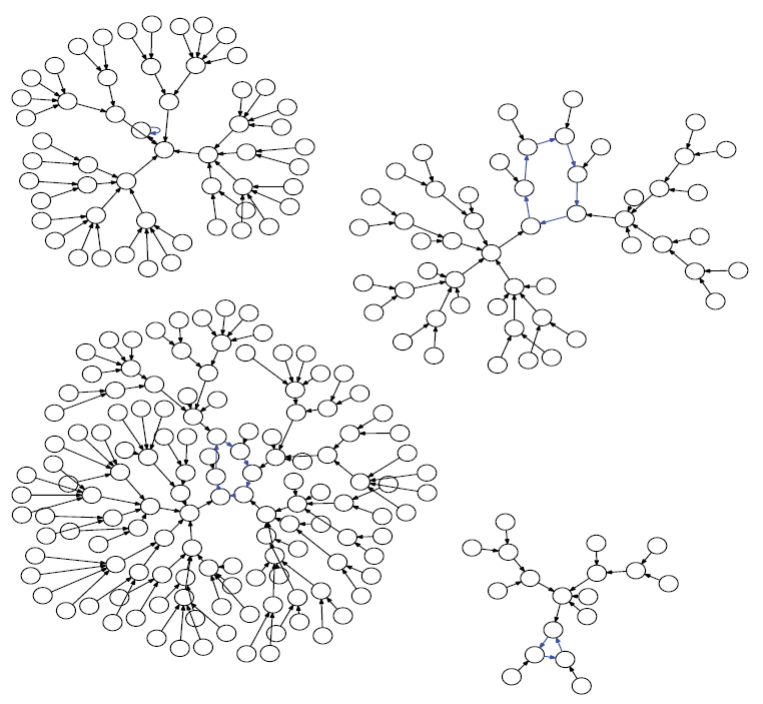
Minimally heterogeneous size 8 XOR+Rule 1.

**Figure 16:**
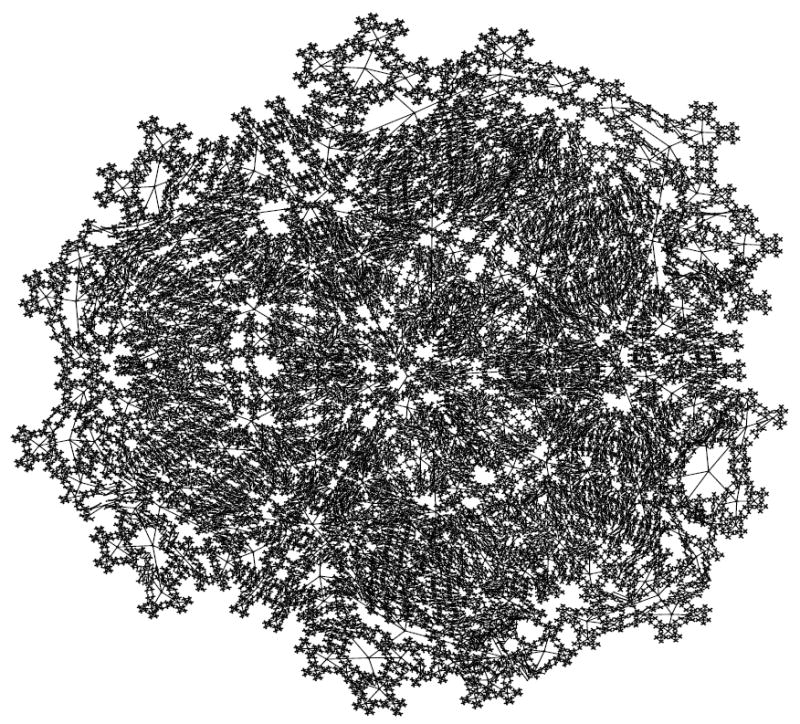
XOR organism size 16.

**Figure 17:**
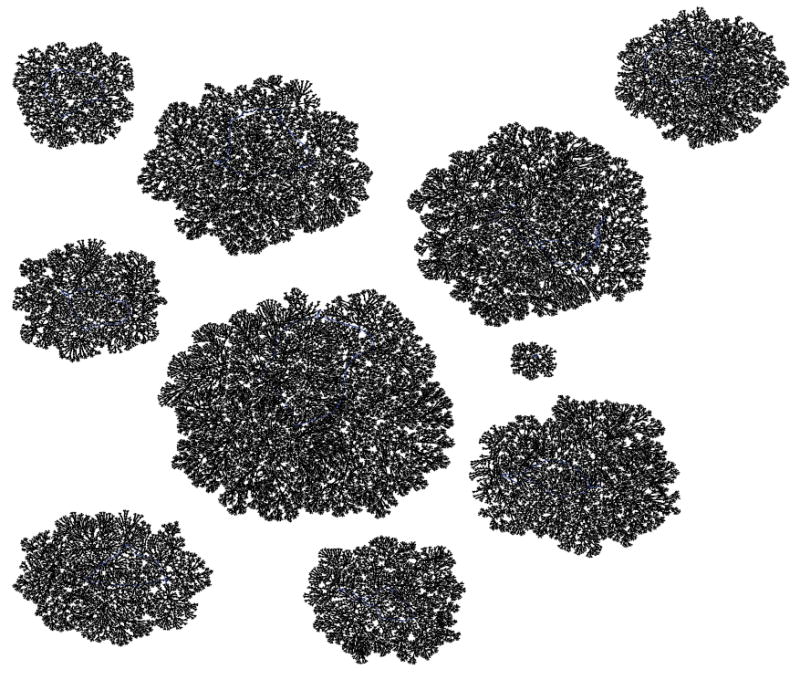
Minimally heterogeneous size 16 XOR+Rule 1.

In Figures 10-13 we show the state space dynamics of minimally heterogeneous organisms at sizes 2, 4, 8 and 16 where the deviant cell applies rule 1 (instead of XOR) to determine its value. For comparison the state space dynamics of the homogeneous organism (in which all cells apply the XOR update rule) of corresponding sizes, can be seen in Figures 14-17. We generate Figures 10-13 as described earlier using experimentally simulated dynamics rendered by Graphviz.

## Low Adaptivity and Organisms of Size 2*^i^*

Our objective is to prove two theorems.

Theorem 1, if the number of cells in an organism is a proper power of 2, then the organism has exactly one attractor, which has length 1 and consists of the state where all cells have a value of 0.

Theorem 2, if regardless of initial state *X*, the organism always ends up in the same attractor, then the number of cells in the organism is a power of 2.

It will take some work to get the proofs of the above statements; they appear in finality on pp. 19. We begin with some definitions in Section 3.1 below. Then, in Section 3.2, we develop a theory of decompositions, which are useful to carry out arguments about the dynamics of the organism’s state over time. In Section 3.3, this is used to prove results about infinite organisms (in which the cells exist on an infinite line). In Section 3.4, these results are wrapped into or “mapped down” to the dynamics of finite cyclic organisms.

### Definitions

#### Structure

We consider organisms whose cellular structure may be modelled as an undirected cyclic graph *C*=(*V*, *E*) of size *n*, whose vertices are considered “cells” and are enumerated *V*={*v*_0_ ,…, *v*_n-1_ }. Each cell *v_i_* in *V* is connected in cyclic order to two neighbors, so that E={*v_i_*, *v*_*i*+1_ (mod n)) | *i*=0,…, *n*-1}. Microscopic cellular behavior within an organism is modeled by fixing a function *f : V* → *F* that assigns to each cell *v* ∈ *V* , a function *f* (*v*) from *F*= {*g* : {0, 1} × {0, 1} → {0, 1}}, the set of all binary Boolean functions; note that |*F*|=2^2•2=^16. The action of *f* at a vertex *v_i_* can be thought of as a truth table mapping *v_i_*’s left and right neighbors’ current state, to *v_i_*’s state at the next time step.

In Table 3 since each of the bits *b*_0_, *b*_1_, *b*_2_, *b*_3_ must be either 0 or 1, in what follows, we will frequently use the 4-bit binary string *b*_0_, *b*_1_, *b*_2_, *b*_3_ to name the function *f*. Together, the pair (*C*, *f*) define the microscopic structure of the organism. An organism is said to be homogeneous if |*Im*(*f*)|=1; otherwise it is said to be heterogeneous.

#### State

Since at each instant, a cell can have a value of either 0 or 1, the instantaneous state of the organism is specifiable as a function V → {0, 1}. The state of the organism over (discrete) time may then be represented by a function *s: V* × *N*→ {0, 1} where *s*(*v_i_*, *t*) is the state of cell *v_i_* ∈*V* at time *t*. Since cell *v_i_* behaves (across all time) according to function *f* (*v*_i_), and all cells are assumed to operate synchronously, the state of the organism evolves over time according to the following law:

$$s(v_{i},t+1)=f(v_{i})\;(s(v_{i-1(\mathrm{mod}\;n)},t),\;s(v_{i+1(\mathrm{mod}\;n)},t))$$

For each *i*=0,… *n*-1 and *t* ≥ 0. Informally, the state of the organism’s constituent cells evolves according to the rule specified by Boolean function operating at that cell, together with the current state of its two adjacent cellular neighbors. We denote the subset of cells whose state is “on” (i.e., 1) at time *t* as *s*+ (*t*)={*v* ∈ *V* | *s*(*v*, *t*)=1}. Note that to identify the system’s state it suffices to know *s*^+^(*t*), since we can infer that the remaining cells are in state 0. In what follows, we will frequently identify the state of the organism at time t with the subset *s*^+^(*t*) ⊂ *V* [8].

### Decompositions

The results presented in this section consider countably infinite populations of cells arranged in an infinite line. We will show that it is always possible to decompose the cells into independent segments, on which the successor function acts independently. One can thus compute the action of the successor function on the organism as a whole by amalgamating its action on each of the independent segments in the decomposition. This is the essential content of the final result in this section, Lemma 10 on pp. 16. Next, in Section 3.3 (pp.17), we use the decompositions to prove significant results about the dynamics of infinite linear organisms. We begin with the following definition (Table 4).

#### Definition 1

Let (Z/2Z)^z^ be the set of functions from the integers Z to the two-element set (Z/2Z= {0, 1}. Each function *x*: Z → {0, 1} in (Z/2Z)^z^ may be represented as an indexed string where the constituent binary symbols are annotated with subscripts from the function’s domain Z.

For example, if *x* is a function which maps the three integers 0, 1 and 7 all to 1 while mapping all other integers to 0, then we will write *x* as a subscripted string, as follows:

$$X=\overset{\leftarrow }{0}1_{0}1_{1}0_{2}0_{3}0_{4}0_{5}0_{6}1_{7}\vec{0}.$$

Here 0⃖ represents an abbreviation for the left-infinite sequence of 0s (for subscripts decreasing to -∞), while 0⃗ is an abbreviation that stands for the right-infinite sequence of 0s (for subscripts increasing to +∞). The bijective correspondence between subscripted strings and functions is unambiguous. Abusing the notation, we denote both the function that is everywhere 0, and it’s associated indexed string, as *Ŝ*.

In the discussion that follows, we shall frequently move back and forth between functions and their indexed string representations. We will adhere to a convention wherein functions in (Z/2Z)^z^ shall be denoted by lowercase letters (e.g., *x*, *y*, *z*) while their bi-infinite binary string representations shall be denoted with the corresponding uppercase letters (e.g., *X*, *Y*, *Z*).

The next definition captures the fact that each individual responds uniformly to the presence/absence of local belief diversity, since XOR (and its negation) are the only two non-constant symmetric Boolean-valued functions on two inputs.

#### Definition 2

Let ⨁ be a binary operator on (Z/2Z)^z^ defined as follows. Given two functions x, y in (Z/2Z)^Z^, the value of (*x* ⨁ *y*):(Z/2Z)^Z^ at integer *i* in , is defined in terms of the exclusive-or ⨁ operation (*x* ⨁ *y*)(*i*)=*x*(*i*) ⨁ *y*(*i*),

Where, the truth table for the ⨁ operation is enumerated in Table 4.

We use Table 4 to define the successor function *Ŝ*, which describes the state of the entire system at each successive time step by applying the XOR update rule synchronously at each constituent cell. For example, if *X* = 0⃖1_0_1_1_0_2_0_3_0_4_0_5_0_6_0_7_0⃗. then *Ŝ X* = 0⃖1_−1_0_0_1_1_0_2_0_3_0_4_0_5_0_6_1_7_1_8_0⃗.

We intend to quantify the properties of *Ŝ* using decompositions (see Definitions 11 and 12), but first we must introduce some notations and preliminary results; this is the objective of Definitions 3-10 and Lemmas 3-8 (on pp. 12-15), which follow.

#### Definition 3

Let *Ŝ* : (Z/2Z)^Z^ → (Z/2Z)^Z^ be a unary operator defined such that for each function *x* in (Z/2Z)^z^ , the value of *Ŝ x* : Z→ Z/2Z at *i* in Z is taken to be *Ŝ x*(*i*)=*x*(*i*-1) ⨁ *x*(*i*+1).

As is customary notation for successive powers of operators, we define *Ŝ*^0^ to be the identity map on (Z/2Z)^Z^ and then inductively put *Ŝ^j^* = *Ŝ* ∘ *Ŝ*^*j*-1^, for each *j* > 0. The successor function *Ŝ* and ⨁ enjoy a close relationship, as Lemmas 3 and 5 make evident.

#### Lemma 3

For all *x*, *y* in (Z/2Z)^Z^, and all *i* ⨁ Z, *Ŝ* (*x* ⨁ *y*)(*i*)= *Ŝ x*(*i*) ⨁ *Ŝ y*(*i*).

#### Proof

By Definitions 2 and 3, we know that

$$\hat{S}\;x(i)⨁\hat{S}\;y(i)=(x(i-1)⨁x(i+1))⨁(y(i-1)⨁y(i+1))$$

$$\hat{S}\;(x⨁y)(i)=(x(i-1)⨁y(i-1))⨁(x(i+1)⨁y(i+1)).$$

The right hand sides of the above equations are equal by the associativity and communicativity of the exclusive-or operation ⨁ over Z/2Z, and thus so are the left-hand sides. The Lemma follows. The previous Lemma suggests that the associative and communicative properties of ⨁ could be leveraged if a function x can be decomposed into a sum (w.r.t ⨁), since the action of *Ŝ* to then be distributed over summands. This idea shall be brought to fruition in Lemma 10.

#### Definition 4

Two functions *x*, *y* ∈ (Z/2Z)^z^ are said to be shift-related, denoted *x* ≈ *y*, if there exists a shift *t* ∈ Z such that *x*(*i*)=*y*(*i*+*t*) for all *i* in Z.

For example, if *X* = 0⃖1_0_0_1_0_2_1_3_0_4_0_5_0_6_1_7_0⃗ and *Y* = 0⃖1_0_0_1_1_2_1_3_0_4_1_5_0_6_1_7_0, then *x*(*i*)=*y*(*i*+*t*) where *t*=1 (for all *i* in Z), and hence *x* and *y* are said to be shift-related. On the other hand, if Z= 0⃖1_0_0_1_1_2_1_3_0_4_1_5_0_6_1_7_0⃗, then *z* is not shift-related to *x*, since there is no integer *t* such that *z*(*i*)=*x*(*i*+*t*) for all *i* in Z. From this it follows that *z* is also not shift-related to *y* which is a specific application of the next Lemma.

#### Lemma 4

The shift-relation ≈ is an equivalence relation on (Z/2Z)^Z^.

#### Proof

Consider functions *x*, *y*, *z* ∈ (Z/2Z)^Z^. Reflexivity is obvious since *x* ≈ *x* by taking *t*=0 in definition 4. If *x* ≈ *y* by shift t, then *y* ≈ *x* by shift −*t*, implying symmetry. Finally, transitivity holds since if *x* ≈ *y* by shift *t*_1_, and *y* ≈ *z* by shift *t*_2_ then *x* ≈ *z* by shift *t*_1_+*t*_2_.

Informally, if two functions are shift equivalent then the results of their successors are also shift equivalent. This is clear from the example strings *X* and *Y* in Definition 4: *Ŝ X*= 0⃖1_−1_0_0_1_1_1_2_0_3_1_4_0_5_1_6_0_7_1_8_0⃗ and where *Ŝ x_i_* = *Ŝy*_*i*+1_ therefore *Ŝ X* ≈ *Ŝ* $\overset{\overset{\leftarrow }{0}1_{0}0_{1}1_{2}1_{3}0_{4}1_{5}0_{6}}{Y}$. The next Lemma proves the general case.

#### Lemma 5

If *x*, *y* ∈ (Z/2Z)^Z^ and *x* ≈ *y*, then *Ŝ x* ≈ *Ŝ y*.

#### Proof

If *x* ≈ *y*, then by Definition 4, there exists *t* ∈ Z such that *x* (*i*)=*y* (*i*+*t*) for all *i* in Z. By definition 3, we know that *Ŝ x*(*i*)=*x*(*i*-1)⨁*x*(*i*+1) and *Ŝ y*(*i*+*t*)=*y*(*i-*1+*t*) ⨁ *y*(*i*+1+*t*). Appealing again to definition 4, we see that *Ŝ x* (*i*) = *Ŝ y* (*i*+*t*), from which it follows that *Ŝ x* ≈ *Ŝ y*.

We shall use an ordinary, non-indexed string representation for ≈-equivalence classes of functions in (Z/2Z)^Z^. Towards this, we introduce the next definition.

#### Definition 5

For each function *x* ∈(Z/2Z)^Z^, let ${\bar{X}}^{\mathrm{def}}=\ldots x(-2).x(-1).x(0).x(1).x(2)\ldots$ be the associated bi-infinite binary (ordinary, non-indexed) string. While definition 1 reflects the fact that every function *x* in (Z/2Z)^Z^ corresponds unambiguously to an indexed string, the next Definition and Lemma capture the fact that this correspondence is not 1-1 in the case of the ordinary non-indexed strings presented in Definition 5.

#### Definition 6

Associated with every bi-infinite binary (ordinary, non-indexed) string *X̄* is a countably infinite 1-parameter family of functions [*X̄*]⊂(Z/2Z)^Z^, wherein ${\bar{X}}^{\mathrm{def}}=\{x_{t}:\mathbb{Z}\to 2\mathbb{Z}|t\in \mathbb{Z}\}$, where $x_{t}{\left(i\right)}^{\mathrm{def}}=x(t+i)$ for all *i* in Z.

#### Lemma 6

For any bi-infinite binary (ordinary, non-indexed) string *X̄* the set [*X̄*] ⊂ (Z/2Z)^Z^ is closed under shift equivalence; that is, (*i*) if , *x_a_* , *x_b_* ∈ [*X̄*] then *x_a_* ≈ *x_b_* and (ii) if *x_a_* ∈ [*X̄*] and *x*_a_ ≈ *y* then *y*∈ [*X̄*].

#### Proof

To see (i) consider two functions *x_a_*, *x_b_* ∈ [*X̄*]. By Definition 6 we know that *x_a_* (*i*)=*x* (*a*+*i*)=*x* (*b*+*i+*(*a*-*b*))=*x_b_* (*i*+(*a-b*)), and so it follows that *x_a_* ≈ *x_b_* by considering a shift of *t*=*a-b* in Definition 4. To see (ii) suppose *x_a_* ≈ *y* for some *x_a_* ∈ [*X̄*] and some *y* ∈ (Z/2Z)^Z^. Then by Definition 4, there exists *t* such that *x_a_* (*i*)=*y* (*i*+*t*) for all *i* in Z, and thus *y* ≡ *x_a_* + *t*, implying that *y* ∈ [*X̄*] by Definition 6. The set *F* of all binary valued functions having finite support (i.e., which take value 1 at only finitely many integers) shall turn out to be of special interest.

#### Definition 7

Let *F* ⊂ (Z/2Z)^Z^ be the set of binary-valued functions on Z having finite support; that is, *x* ∈ *F* if *x* (*i*)=0 for all but finitely many *i* ∈ Z. For example, $b(x)=\left\{ \begin{array}{cc} \min\{i|x\;(i)=1\} & x\neq 0\\ \;0 & x=\bar{0} \end{array}\right.$ corresponds to a function *x* that lies in *F*, since *X* contains only three 1s. On the other hand, a function *x*′ which sends all even integers to 1 and all odd integers to 0, lies in (Z/2Z)^Z^\*F*. For functions of finite support, it will frequently be useful to refer to the least and greatest integer which map to 1. Towards this, we introduce the next definition.

#### Definition 8

Let *b*, *e: F* → Z be defined as follows:

$${b(x)}^{\mathrm{def}}=\left\{ \begin{array}{cc} \min\{i|x\;(i)=1\} & x\neq \bar{0}\\ \;0 & x=\bar{0} \end{array}\right.$$

$${e(x)}^{\mathrm{def}}=\left\{ \begin{array}{cc} \max\{i|x(i)=1\} & x\neq \bar{0}\\ \;-1 & x=\bar{0} \end{array}\right.$$

For each function *x* in *F*, the length of |X|^*def*^ = *e*(*x*)−*b*(*x*)+1 is taken to be the number of bits in the largest essentially non-zero subsegment of *X*. Continuing the previous example *X* = 0⃖1_0_0_1_0_2_1_3_0_4_0_5_0_6_1_7_0⃗, we note that *b*(*x*)=0 and *e*(*x*)=7 and |*x*|=8. This suggests that we can “shell” the set F by partitioning it into disjoint subsets and assigning each function *x* ∈ *F* to a specific subset on the basis of |*x*|. The subsequent Definition and Lemma achieves such a shelling.

#### Definition 9

Let *B*_0_ denote the singleton set consisting of the empty string, and for each integer *n* > 0 let *B_n_* denote the set of binary strings beginning and ending in 1 and having of length *n*. Put $B=\cup_{n=0}^{\infty }B_{n}$.

Note that the sets *B_n_* consist of finite ordinary non-indexed binary strings of length *n*. The next Lemma places the set of ≈-equivalence classes of binary functions with finite support into 1-1 correspondence with the set of finite ordinary non-indexed binary strings.

#### Lemma 7

The quotient *F*/≈ is in natural bijective correspondence with *B*.

#### Proof

We map 0̄ ∈ *F* to the empty string in *B*_0_ ⊂ *B* having length 0. It remains to demonstrate a bijection *ϕ* between *F* {0̄} and the set of binary strings of finite positive length which begin and end with 1. Given *x* ∈ *F*, *x* ≠ 0̄, we take *ϕ*(*x*) ∈ *B*_*e*(*x*)-*b*(*x*)+1_ ⊂ *B* to be the string *ϕ*(*x*)=*x*(*b*(*x*)) • *x*(*b*(*x*)+1)• • •*x*(*e*(*x*)-1) • *x*(*e*(*x*)).

Clearly if *x* ≠ *x*′ as functions, then *ϕ*(*x*) and *ϕ*(*x*′) are distinct members of *B*. Moreover, if *y* ∈ *F* and *y* ≈ *x* then *ϕ*(*x*)=*ϕ*(*y*).

In the reverse direction, given a binary string *X* ∈*B_n_* ⊂ B of positive length |*X* |=n>0, we write *X* as a sequence of binary bits having finite positive length

$$X=X_{0}X_{1}•••X_{i}•••X_{n-2}X_{n-1}$$

and consider the function *x* ∈ *F* given by

$$x(i)=\left\{ \begin{array}{cc} X_{i} & 0\leq i<|X|\\ 0 & \mathrm{otherwise}. \end{array}\right.$$

Since *X* has positive length, *X* ≠*Y* , and *ϕ*^-1^ (*X*) is taken to be the ≈-equivalence class of *x*. Clearly if *Y* ∈ *B* and *X* ≠*Y* , then *ϕ*^-1^ (*X*) ∩ *ϕ*^-1^ (*Y*)= ∅.

#### Definition 10

By Lemma 5, the operator *Ŝ* factors through the ≈ relation, and thus the action of *Ŝ* on *F* ⊂ (Z/2Z)^Z^ presented in Definition 3 induces an operator (which we shall denote as *S*) on the quotient set *F* / ≈. Since *F* / ≈ was shown to correspond to the set *B* in Lemma 7, we arrive at an induced unary operator *S: B* → *B*. The function *S* is thus a self-map of *B*, which is a set of strings that contains all finite strings beginning and ending with 1 (as well as the empty string).

With the preceding definitions in hand, we return to the evolution of the dynamical systems over time under the action of the successor function *Ŝ*. The next Lemma shows that for functions with finite support, the function’s support interval expands outwards under the action of *Ŝ* ; in particular | *Ŝ x*|=|*x*|+2.

#### Lemma 8

Let x ∈ *F*\ {*Ŝ*}. Then

$$\begin{array}{l} b(\hat{S}\;x)=b(x)-1\\ e(\hat{S}\;x)=e(x)+1 \end{array}$$

#### Proof

Since *x*(*b*(*x*))=1 and *x*(*b*(*x*)-2)=0, by Definition 3, *Ŝ x*(*b*(*x*)-1)=1 and since for all *i* < *b*(*x*)-1, *x*(*i*-1)=*x*(*i*+1)=0, it follows that b(*Ŝ x*)=*b*(*x*)-1. Analogously, since *x*(*e*(*x*))=1 and *x*(*e*(*x*)+2)=0, by Definition 3, *Ŝ x*(*e*(*x*)+1)=1 and since for all *i* > *e*(*x*)+1, *x*(*i*-1)=*x*(*i*+1)=0, it follows that *e*(*Ŝ x*)=*e*(*x*)+1.

Given a function *x* ∈ *F*, we can decompose its string representation *X* into c disjoint component strings, where each of the components has 0r as a prefix and suffix. Such decomposition shall be useful to factor the action of *Ŝ*^r^ on *x* into a set of independent action on each of the *c* components. The Definition below renders the decomposition formally.

#### Definition 11

Given a function *x* ∈ *F*, integers *r ≥* 0 and *c* ≥ 1. Choose *r_j_* > 0 and *g_j_* ≥ 0 (for *j*=1 ,…, *c*), and let *P* be a partition of the set {*b*(*x*)-*r*_1_ ,…, *e*(*x*)+*rc* } ⊆ Z

$$P=\{(b_{1},b_{1}+1,\ldots ,e_{1}),(b_{2},b_{2}+1,\ldots ,e_{2}),\ldots ,(bc,bc+1,\ldots ,ec)\}$$

into *c* contiguous integer subsequences, in a manner which additionally satisfies:

1. *b*_1_=*b*(*x*)-*r*_1_; *ec*=*e*(*x*)+*r_c_*
2. For *j*=1 ,…, *c*:
  - e_*j*_≥ *b*_*j*+_ 2r_*j*_;
  - *x*(*b*_*j*+_*r_j_*)=*x*(*e_j_*-*r_j_*)=1;
  - For all *i* satisfying *b_j_* ≤ *i* < *b*_*j*+_ *r_j_* or *e*_*j*-_ *r_j_* <*i* ≤ *e_j_*, *x*(*i*)=0.
3. b_j+1=_e_j_+g_j_+1, for all j=1 ,…, c − 1.

Then, for *j*=1,…, *c*, define

$${{\tilde{W}}^{j}}^{\mathrm{def}}=x(b_{j}+r_{j})\cdot x(b_{j}+r_{j}+1)\cdots x(e_{j}-r_{j}-1)\cdot x(e_{j}-r_{j})$$

and take ${W^{j}}^{\mathrm{def}}=0^{\mathrm{rj}}\cdot {\tilde{W}}^{j}\cdot 0^{\mathrm{rj}}$. Note that ${\tilde{W}}^{j}\in B_{e_{j}-b_{j}-2rj+1}$. Take $r=\min_{j=1}^{c}\{r_{j}\}$

We refer to the tuple (*b*_1_, *W*^1^, *g*_1_ , *W*^2^, *g*_2_ ,…, *g*_c-1_ , *W*^c^) as an (*r, c*)-decomposition of *x*.

To compute, for example, a (1, 3) decomposition of our ongoing example *X* = 0⃖1_0_0_1_0_2_1_3_0_4_0_5_0_6_1_7_0⃗, we need to provide a size 3 partition of the sequence (−1, 0, 1 ,…, 7, 8) into contiguous integer sequences. If we take *P*={(−1, 0, 1), (2, 3, 4), (6, 7, 8)}, then conditions 1-3 of Definition 11 can be verified directly, noting that *b*_1_=−1, *e*_1_=1, *g*_1_=0, *b*_2_=2, *e*_2_=4, *g*_2_=1, *b*_3_=6, *e*_3_=8; note that *g*_2_ maintains the gap between the sub-segments of indices (2, 3, 4) and (6, 7, 8). It follows that *X* = 0⃖1_0_0_1_0_2_1_3_0_4_0_5_0_6_1_7_0⃗ is a (1, 3) decomposition of X.

The structure of (*r,c*)-decompositions factor through the equivalence relation ≈. For example, referring to the strings *X* = 0⃖1_0_0_1_0_2_1_3_0_4_0_5_0_6_1_7_0⃗ and *Y* = 0⃖1_1_0_2_0_3_1_4_0_5_1_6_0_7_1_8_0⃗ introduced subsequent to Definition 4, we see that $\left(\bar{010},0,\bar{010},1,\bar{010}\right)$ is a (1, 3)-decomposition of *X*, while $\left(\bar{010},0,\bar{010},1,\bar{010}\right)$ is a (1, 3)-decomposition of *Y*. The fact that Y is a *t*=1 shift of *X* is reflected in the fact that *b*_1_=0 decomposition of *Y*, a value that is 1 greater than its value in the decomposition of *Y*. This observation is stated formally below:

#### Lemma 9

Let *X* ∈ *B*, and *x*, *x*′ ∈ [*X*]. Let *t* be an integer for which *x*′ (*i+t*)=*x*(*i*) for all *i* ∈ Z. If (*b*_1_, *W*^1^, *g*_1_, *W*^2^, *g*_2_,…, *g*_c-1_, *W*^c^) is an (*r, c*)-decomposition of *x*, then (*b*_1+t_, W^1^, g_1_, W^2^, g_2_,…, g_c-1_, W^c^) is an (*r, c*)-decomposition of *x*′.

The above allows us to extend the definition of (*r, c*)-decompositions to ≈-equivalence classes of functions.

#### Definition 12

For each *X* ∈ *B*, take *x* ∈ [*X*] and let (*b*_1_, *W*^1^, *g*_1_, *W*^2^, *g*_2_ ,…, *g*_c-1_, *W*^c^) is an (*r, c*)-decomposition of *x*. We refer to the tuple (*W*^1^,*g*_1_, *W*^2^, *g*_2_,…, *g*_c-1_, *W*^c^) as an (*r, c*)-decomposition of the ≈-equivalence class [*X*].

Continuing our example, $\left(\bar{010},0,\bar{010},1,\bar{010}\right)$ is a (1, 3)-decomposition of [*X*]=[*Y*]. We note by definition each (*r, c*)-decomposition (*b*_1_, *W*^1^, *g*_1_, *W*^2^, *g*_2_,…., *g*_c-1_, *W*^c^) of *x* ∈ *F* gives rise to a set of functions *x_j_*: (Z/2Z)^Z^ (for *j*=1,…,*c*) where,

$$x_{j}(i)=\left\{ \begin{array}{cc} x(i) & b_{j}\leq i\leq e_{j}\\ 0 & \mathrm{otherwise}. \end{array}\right.$$

Satisfying the relation *x*=*x*_1_ ⨁ *x*_2_ ⨁ … ⨁ *x*_c_. This identity quantifies the manner in which we decompose *x* into a ⨁ sum, each summand of which may be seen as being acted upon independently by *Ŝ*.

#### Lemma 10

Given *X* ∈ *B*, let (*W*^1^, *g*_1_, *W*^2^, *g*_2_,…, *g*_c-1,_ *W*^c^) be an (*r, c*)-decomposition of [*X*], for fixed integers *r* ≥ 0 and *c* ≥ 1. Then for each integer 0 < *t* ≤ *r*, the tuple

$$\left(0^{r-t}\cdot S^{t}{\tilde{W}}^{1}\cdot 0^{r-t},g_{1},0^{r-t}\cdot S^{t}{\tilde{W}}^{2}\cdot 0^{r-t},g_{2}\ldots ,g_{c-1},0^{r-t}\cdot S^{t}{\tilde{W}}^{c}\cdot 0^{r-t}\right)$$

is an (*r-t*, *c*)-decomposition of [*S^t^X*].

#### Proof

Fix *x* ∈ [*X*] and let (*b*_1_,*W*^1^, *g*_1_ , *W*^2^, *g*_2_ ,…, *g*_c−1_, *W*^c^) be an (*r, c*)-decomposition of *x*. For *j*=1 ,…, c by Definition 11, we know that *x*(*b*_*j*+_*r*)=*x*(*e_j_*-*r*)=1, and *x*(*i*)=0 whenever *b_j_* ≤ *i* < *b*_*j*+_ *r* or *e_j_*-*r* < *i* < *e_j_*. Moreover,

$$W^{j}=\underset{{\tilde{W}}^{j}}{\underset{︸}{0^{r}\cdot x(b_{j}+r)\cdot x(b_{j}+r+1)\cdots x(e_{j}-r-1)x(e_{j}-r)\cdot 0^{r}}}$$

Viewing *W^j^* → *X* as a substring, by Lemma 8 we see that *b*(*Ŝ*^t^ *x*)=*b*(*x*)-*t*, and *e*(*Ŝ*^t^ *x*)=*e*(*x*)+*t*. Since (by assumption) *t* ≤ *r*, it follows that

$$\left(b_{1}-t,0^{r-t}S^{t}{\tilde{W}}^{1}\cdot 0^{r-t},g_{1},0^{r-t}\cdot S^{t}{\tilde{W}}^{2}\cdot 0^{r-t},g_{2}\ldots .,g_{c-1},0^{r-t}\cdot S^{t}{\tilde{W}}^{c}\cdot 0^{r-t}\right)$$

is an (*r*-*t*, *c*)-decomposition of *Ŝ^t^ x*. The conclusion of the Lemma follows by taking the above (*r*-*t*, *c*)- decomposition of Sˆt *x* and considering it as the basis of an (*r, c*) decomposition of the ≈-equivalence class [*S^t^X*], as per Definition 12.

The previous Lemma demonstrates that (*r, c*)-decompositions are a parsimonious way of describing the action of *Ŝ* on *x* ∈ *F* as an aggregation of separate independent actions of *S* smaller sub-segments of *X*. This will be useful repeatedly in the arguments that follow.

### The infinite case

The main theorem of this section is the formal proof of the assertion that if you start with a state that consists of just two 1s separated by some number of zeros, and then simulate forward, you will again at some point enter a state that has just two 1s separated by (an even larger) number of zeros. More precisely, if you start with two 1s separated by 2^*i*^-1 zeros, then after 2^*i*^−1 steps, you will arrive at a state where you have two 1s separated by 2^*i*^+1-1 zeros. Next, in Section 3.4 (pp. 19), we use this theorem to prove important results about the dynamics of finite cyclic organisms.

Formally stated:

#### Theorem 11

$$\forall i\geq 2\;S^{2^{i-1}}{(10}^{2^{i}-1}1)=10^{2^{i}-1}1$$

Recalling *S: B* → *B* from Definition 10, we introduce the following named assertion *ϕ*:

#### Definition 13

For fixed integer *i* ≥ 2, put

$$\phi_{i}:S^{2^{i-1}-1}{(10}^{2^{i}-1}1)={\left(10\right)}^{2^{i}-1}1$$

The main result proved in this section (Proposition 18) is that for all *i* ≥ 2, assertion *ϕ_i_* is true. This proof shall proceed by induction, for which the next Lemma provides the base case.

#### Lemma 12

*ϕ*_2_ is true.

#### Proof

It suffices to show *S*^1^(10^3^1)=(10^3^)1. Noting that $\left(\bar{00100}\right)$ is a (1,1)-decomposition of [10^3^1], by Lemma 10 we know (*S*^1^(10^3^1)) is a (0, 1)-decomposition of [*S*^1^(10^3^1)], and since *S*^1^(10^3^1)=1010101=(10^3^)1, the assertion is proved.

#### Lemma 13

*S*^2^(1)=10^3^1.

#### Proof

Noting that $\left(\bar{00100}\right)$ is a (2, 1)-decomposition of [1], by Lemma 10 we know (*S*^2^(00100)) is a (0,1)-decomposition of [*S*^2^(1)], and since *S*^2^(00100)=10001=10^3^1, the assertion is proved.

#### Lemma 14

For all *k* ≥ 1, *S*^1^((10)^*k*−1^1)=10^2*k*−1^1.

#### Proof

Since $\left(\bar{0{\left(10\right)}^{k-1}10}\right)$ is a (1, 1)-decomposition of [(10)^*k*−1^1], by Lemma 10 we know (0•*S*^1^ ((10)^*k*−1^1)•0) is a (0, 1)-decomposition of [*S*^1^((10)^*k*−1^1)], and since *S*^1^((10)^*k*−1^1)=10^2*k*−1^1, the assertion is proved.

#### Lemma 15

If ∀*i* > *j* ≥ 2, *ϕ*_j_ is true, then $S^{2^{i-1}}{(10}^{2^{i}-1}1){=}_{S^{2^{i-1}-1}{(10}^{2^{i}-1}1)=S^{1}(S^{2^{i-1}-1}(10^{2^{i-1}-1}1))}$

#### Proof

First we write $S^{2^{i-1}-1}{(10}^{2^{i}-1}1)=S^{1}(S^{2^{i-1}-1}(10^{2^{i-1}-1}1))$. Now, by the inductive hypothesis:

$$S^{2^{i-1}-1}{(10}^{2^{i}-1}1)={\left(10\right)}^{2^{i}-1}1$$

By appealing to Lemma 14 we $S^{1}{\left((10\right)}^{2^{i}-1}1)=10^{2^{i+1}-1}1$, which completes the proof.

#### Lemma 16

If ∀*i*<*x*, *ϕ_i_* is true, then 0<*k*<*x* implies $S^{2^{k}-3}{(10}^{3}1)={\left(10\right)}^{2^{k}-1}1$.

#### Proof

We begin by noting that

$$2^{k}-3=(2^{k-1}-1)+(2^{k-1}-2)=(2^{k-1}-1)+\sum_{j=1}^{k-2}2^{j}$$

Thus,

$$S^{2^{k}-3}{(10}^{3}1)s^{2^{k-1}-1}\circ s^{2^{k-2}}\circ s^{2^{k-3}}\cdots s^{2^{2}}\circ s^{2^{1}}{(10}^{2^{2}-1}1)$$

Repeated application of Lemma 15 yields

$$\begin{array}{c} S^{2^{1}}{(10}^{2^{2}-1}1)=10^{2^{3}-1}1\\ S^{2^{2}}{(10}^{2^{3}-1}1)=10^{2^{4}-1}1\\ S^{2^{3}}{(10}^{2^{4}-1}1)=10^{2^{5}-1}1\\ \ldots \\ S^{2^{k-2}}{(10}^{2^{k-1}-1}1)=10^{2^{k}-1}1 \end{array}$$

It remains to compute $S^{2^{k-1}-1}{(10}^{2^{k}-1}1)$. Since *k*<*x*, we may assume the inductive hypothesis: *ϕ*_k_ is true. From this it follows that $S^{2^{k-1}-1}{(10}^{2^{k}-1}1)=10^{2^{k}-1}1$.

#### Lemma 17

If *ϕ_i_* is true ∀_*i*_ <*x* then *ϕ_x_* is true.

#### Proof

It suffices to show: $S^{2^{x-1}-1}{(10}^{2^{x}-1}1)=10^{2^{x}-1}1$. We begin by noting that

$$10^{2^{x-1}-1}{1=10}^{2^{x}/2}\cdot 0^{2^{x}/2-1}1=10^{2^{x-1}}1\cdot 0^{2^{x-1}-1}1$$

and thus

$$10^{2^{x-1}-1}{1=10}^{2^{x}/2}\cdot 0^{2^{x}/2-1}1=10^{2^{x-1}}1\cdot 0^{2^{x-1}-1}1$$

is a (2^*x*−1^ 1, 2)-decomposition of $10^{2^{x-1}-1}$. So, by Lemma 10

$$\left(S^{2^{x-1}-1}(0^{2^{x-1}-1}10^{2^{x-1}-1}),0,S^{2^{x-1}-1}(0^{2^{x-1}-1}10^{2^{x-1}-1})\right)$$

is a (0, 2)-decomposition of [ $S^{2^{x-1}-1}(10^{2^{x}-1}1)$]. Using Lemma 13, $S^{2^{x-1}-1}$ (1) may be re-expressed as:

$$S^{2^{x-1}-1-2}\circ S^{2}(1)=S^{2^{x-1}-1-2}{(10}^{3}1)$$

Appealing to Lemma 16 we determine thatm $S^{2^{x-1}-1-2}{(10}^{3}{1)=(10)}^{2^{x-1}-1}1$. Thus, the (0, 2)-decomposition of [ $S^{2^{x-1}-1}{\left(10\right)}^{2^{x-1}-1}1$] is in fact $\bar{{\left((10\right)}^{2^{x-1}-1}1},1,\bar{{\left(10\right)}^{2^{x-1}-1}1)}$.

Concatenating the two factors and the intervening zero (since *g*_1_=1), we conclude that $\bar{{\left((10\right)}^{2^{x-1}-1}1)}$ is a (0, 1)-decomposition of [ $S^{2^{x-1}-1}{\left(10\right)}^{2^{x-1}-1}1$] . The assertion is proven.

The proof of Proposition 18 is now immediate.

#### Proposition 18

For all *i* ≥ 2,*ϕ*_i_ is true.

#### Proof

The base case is given by Lemma 12, and the inductive step by Lemma 17.

We are now ready to prove Theorem 11.

Proof, directly from Lemma 15 where *i* ≥ 2, applying Proposition 18 shows that *ϕ_i_* is true for all *i* ≥ 2.

### Going from infinite to finite

Suppose now that instead of operating with infinite strings (functions on Z), the operation is taking place on a cycle of *N* cells numbered 0, 1,…, *N*−1, each of which could take a value of 0 or 1.

#### Lemma 19

If *X*=0*^N^* then *S*(*X*)=0*^N^*

#### Proof

This is by definition of the XOR function. Any cycle in which all cells have the value 0 will remain unchanged over time, that is S(0*^N^*)=0*^N^*.

#### Lemma 20

If there is one attractor, then the attractor is 0*^N^*.

#### Proof

Suppose we have one attractor. Because an initial state X=0*^N^* is possible by definition of the networks, applying Lemma 19 completes the proof.

#### Definition 14

A state *X* is said to lead to a state *Y* denoted as X → Y if ∃k such that *S*^k^(*X*)=*Y*.

We are now ready to prove Theorem 1 (stated originally on pp. 8): If the number of cells in an organism is a proper power of 2, then the organism has exactly one attractor, which has length 1, and consists of the state where all cells have a value of 0.

#### Proof

Suppose *N* is a power of 2. Consider the starting state 0^N-1^ 1. By Lemma 8, simulating forward from this start state produces a wave of non-zero values expanding outwards along the cycle from cell 0. The two wave frontiers proceed in opposite directions, eventually colliding on the cycle’s topology at cell N/2 that is antipodal to cell 0. By combining Lemma 13 and Lemma 16 we see that, $S^{2^{i-1}-1}{\left(1)=(10\right)}^{2^{i-1}-1}1$, a string of length 2*^i^*−1. Thus, at discrete time step 2^*i*−1-^1, the cells of the cycle are in state: ${11:10}^{2^{i+1}-1}1$, implying that cells strictly alternate as 0, 1, 0, 1,… in their value. Now at this time, because all cells witness local homogeneity (that is, for every cell, either both neighbors are 0 or both neighbors are 1), at the next discrete step, all cells in the system take value 0 (since 0 ⨁ 0=1 ⨁ 1=0). Thus, starting from a simple initial state in which precisely one cell has the value 1 and all others have the value 0, we see that the cycle of N=2*^i^* cells converges in 2^*i*−1^ =N/2 discrete time steps to being uniformly 0 everywhere.

Since every complex initial state can be decomposed into an ⨁ sum of simple states by taking one summand for each cell that has the value 1—Lemma 3 can be applied to analyze the evolution of the system from complex states as well. Because every simple initial state converges to the state in which all cells have the value 0 in T=2^*i*-1^ steps, Lemma 3 implies that every complex initial state also converges to the state in which all cells have the value 0 in T=2^*i*-1^ steps. In other words, every initial state X → 0*^N^*, we have shown that the organism has precisely one attractor, namely 0*^N^*

We are also ready to prove Theorem 2 (stated originally on pp. 8): If regardless of initial state *X* the organism always ends up in the same attractor, then the number of cells in the organism is a power of 2.

#### Proof

By applying Lemma 19 and Lemma 3, it suffices to show that for a simple initial state *X^i^*=0^*N* -1^1 where *i* is the index of the cell with the value 1 if *X^i^* → 0*^N^* then for every complex intial state *X* composed of any set of *X^i^* , *X* → 0*^N^*.

Applying Lemma 13, we know *S*^2^(^*N* −1^1)=10^3^ 1. Then, repeatedly apply Lemma 11 so that after each additional 2^*i* -1^ successor steps we have two cells of value 1 separated by 2^*i*+1-^1 cells with value 0. These cells wrap around a cyclic network of *N* cells. 1⨁1=0, by definition of the XOR function. In wrapping the cell values around the network of *N* cells, resulting state would be 0*^N^* if the only two cells with value 1 collide *i*+1 at the same index. For this collision to occur the number of intervening zeros in Lemma 11: $10^{2^{i+1}-1}1$ equal *N*-1 mod *N*.

Therefore, in order for every state 0^*N* -1^1 to lead to the state 0*N* for a network of size *N* the following must be true:

2^*i*+1^−1 ≡ *N*-1 mod *N*

2^*i*+1^≡ 0 mod *N*

In other words, *N* divides 2^*i*+1^.

We have shown that the organism must have size *N*=2*^j^* for some integer *j*.

## Conclusions

In this work, we used an experimental approach to explore the dynamics of homogeneous cyclic organisms of size *N*=2,…,20 cells, using software developed previously [8]. In these homogeneous organisms, each cell synchronously determines its successive states by computing the ⨁ of the value of its neighbors. From observations of these computationally simulated dynamics, we conjectured that if the number of cells in an organism is a proper power of 2, then the organism has exactly one attractor, which has length 1 and consists of the state where all cells have a value of 0. We then formally proved this statement as well as its converse: that if regardless of its initial state the organism always ends up in the same size 1 attractor, then the number of cells in the organism is a proper power of 2. Some of the evidence for this now-proven “if and only if” relationship is rendered in Figures 14, 15 and 16 versus Figures 7 and 8.

Since the act of incrementing the size of an organism until it reaches double its size necessarily requires traversing a number that is a proper power of 2, any organism that grows to more than twice its original size will necessarily encounter a stage in which it has minimal adaptivity and maximal robustness. If the organism seeks to always maintain “intermediate” values of adaptivity and robustness as it grows, then alternative growth patterns that exhibit more than one attractor at powers of 2 sizes will be evolutionarily advantageous. The alternative growth pattern we explore experimentally in this work, is one in which the organism departs from cellular homogeneity to a minimal extent—allowing a single constituent cell to apply a rule that is different from XOR. Through experiments, we show that at sizes that are powers of 2, such minimally heterogeneous organisms avoid manifesting the low adaptivity that is provably exhibited in homogenous organisms. We conclude that cellular differentiation is one way an organism can avoid low adaptivity configurations that would otherwise necessarily be encountered during organism growth. It follows that if there is evolutionary pressure selecting for adaptivity, then the phenomenon of organism growth may express this pressure as a drive towards cellular differentiation and the progression from homogeneity towards heterogeneity. Figures 10-13 show the dynamics graphs of minimally heterogeneous organisms of sizes 2, 4, 8, and 16 respectively, and have increasing numbers of attractors. These figures are placed side by side with the dynamics graphs of homogeneous organism of the same size, to further illustrate their contrasting dynamics.

Future work will entail simulation of more complex growth patterns, beyond merely homogeneous and minimally heterogeneous growth. One pattern we plan to explore is probabilistic cellular differentiation during growth. Future work needs to look at both larger and more diverse organisms, but for larger organisms exhaustive simulation is computationally intractable, requiring advances in random sampling and estimation theory. By considering dynamics data from a more diverse and larger range of systems, it may be possible to identify other evolutionary pressures (beyond cell differentiation) and meta-phenomena that arise as organisms attempt to maintain a balance between adaptivity and robustness during growth.

## Figures and Tables

**Table 1:** Table of update rules.

**Rule 0=**	0
**Rule 1=**	¬*V*_*i*-1_ ∧ ¬*V*_*i*+1_
**Rule 2=**	¬*V*_*i*-1_ ∧ *V*_*i*+1_
**Rule 3=**	¬*V*_*i*-1_
**Rule 4=**	*V*_*i*-1_ ∧ ¬*V*_*i*+1_
**Rule 5=**	¬*V*_*i*+1_
**Rule 6=**	(*V*_*i*-1_ ∧ ¬*V*_*i*+1_) ∨ (¬*V*_*i*-1_ ∧ *V*_*i*+1_)
**Rule 7=**	¬(V_*i*-1_ ∧ V_*i*+1_)
**Rule 8=**	*V*_*i*-1_ ∧ *V*_*i*+1_
**Rule 9=**	(*V*_*i*-1_ ∧ ¬*V*_*i*+1_) ∧ (¬*V*_*i*-1_ ∧ ¬*V*_*i*+1_)
**Rule 10=**	*V*_*i*+1_
**Rule 11=**	*V*_*i*+1_ ∨ (¬*V*_*i*-1_ ∧ ¬*V*_*i*+1_)
**Rule 12=**	*V*_*i*-1_
**Rule 13=**	*V*_*i*-1_∧ (¬*V*_*i*-1_ ∧ ¬*V*_*i*+1_)
**Rule 14=**	*V*_*i*-1_ ∨ *V*_*i*+1_
**Rule 13=**	1

Rule numbers expressed as Boolean logic with 2 inputs: The value of the left neighbor *V*_*i*-1_, and the value of the right neighbor: v_i+1_

**Table 2:** Table of minimally heterogeneous organism attractor count as size increases by powers of 2.

Size	Rule 1	Rule 3	Rule 5	Rule 7	Rule 8	Rule 10	Rule 12	Rule 14
2	1	1	1	1	1	1	1	1
4	1	1	1	1	3	3	3	3
8	4	4	4	4	4	4	4	4
16	10	10	10	10	22	22	22	22
32	≥ 530	≥ 793	≥ 809	≥ 505	≥ 527	≥ 804	≥ 806	≥ 505
64	≥ 998	≥ 1000	≥ 1000	≥ 1000	≥ 998	≥ 1000	≥ 1000	≥ 998
128	≥ 1000	≥ 1000	≥ 1000	≥ 1000	≥ 1000	≥ 1000	≥ 1000	≥ 1000
256	≥ 1000	≥ 1000	≥ 1000	≥ 1000	≥ 1000	≥ 1000	≥ 1000	≥ 1000

**Table 3:** XOR truth table with inputs at time t and resulting output at time *t* + 1.

*s*(*v*_*i*-1_, *t*)	*s*(*v_i_*, *t*)	*s*(*v*_*i*+1_, *t*)	*s*(*v_i_*, *t*+1)
0	*	0	*b*_0_
0	*	1	*b*_1_
1	*	0	*b*_2_
1	*	1	*b*_3_

**Table 4:** Table of XOR truth table.

A	B	A⨁B
0	0	0
0	1	1
1	0	1
1	1	0
